# Apparent Diffusion Coefficient Histogram Analysis of Adenoid Cystic Carcinoma of the Head and Neck: Correlation with Histological Grade and Exploratory Survival Analysis

**DOI:** 10.3390/diagnostics16213452

**Published:** 2026-10-22

**Authors:** Weiqing Tang, Jingbo Wang, Jiliang Ren, Xing Liang, Ying Yuan, Xiaofeng Tao

**Affiliations:** Department of Radiology, Shanghai Ninth People’s Hospital, Shanghai 200011, China; 13817017639@163.com (W.T.); liangxingn1@163.com (X.L.)

**Keywords:** adenoid cystic carcinoma, apparent diffusion coefficient, histogram analysis, histological grade, prognosis

## Abstract

**Background**: In this study, we aimed to evaluate the feasibility of a whole-tumor histogram analysis of apparent diffusion coefficient (ADC) values derived from diffusion-weighted imaging (DWI) for predicting adenoid cystic carcinoma (ACC) histological grade and solid-component status. **Methods**: This retrospective study included 168 patients with primary head and neck ACC who underwent preoperative 3.0 T MRI with DWI, randomly allocated with stratification by grade to a derivation cohort (*n* = 107) and an internal validation cohort (*n* = 61). Whole-tumor ADC histogram features were extracted and reduced through correlation analysis, and the two most discriminative features were combined in a logistic regression model. Overall survival was explored using Cox regression. **Results**: ADC_10_ alone (the 10th percentile of ADC) had areas under the receiver operating characteristic curve (AUCs) of 0.821 (95% confidence interval [CI] 0.727–0.899) and 0.780 (95% CI 0.643–0.895) for grade III in the derivation and validation cohorts, and 0.743 and 0.709 for solid-component status; adding the low-ADC volume did not improve discrimination (DeLong *p* ≥ 0.468). The model output was associated with overall survival in the pooled cohort (hazard ratio per standard deviation, 1.627) but not in the validation cohort or after adjustment for grade. **Conclusions**: ADC_10_ showed promising discrimination of grade III ACC in internal validation, with no gain from adding the low-ADC volume; the prognostic value is unconfirmed, and multicenter external validation is warranted.

## 1. Introduction

Adenoid cystic carcinoma (ACC) is a rare malignancy that primarily originates in the salivary glands. The incidence of ACC is estimated to be approximately 3–4.5 cases per million people [1], accounting for 1% of head and neck cancers and 10% of salivary gland tumors [2]. Despite its rarity, it has been described as “one of the most biologically destructive and unpredictable tumors of the head and neck” [3] due to its characteristics of slow growth, high frequency of local recurrence, propensity for perineural spread, and development of distant metastases [4].

ACCs show three growth patterns, tubular, cribriform and solid, and histological grade, which reflects the proportion of the solid pattern, can serve as an independent prognostic factor [5]. The degree of increase in cellularity from tubular to solid growth pattern is linked to increasingly poor prognosis [5,6,7,8]. Because every specimen has mixed patterns and histologic grades, it is difficult to determine the histological grade using only a single biopsy specimen. Accurate grading is possible only when the entire tumor tissue is available. The process of obtaining an entire tumor can be exacting, time-consuming, and sometimes not feasible due to tumor location [9].

Magnetic resonance imaging (MRI) plays an increasingly vital role in the evaluation of head and neck tumors, owing to its multiple sequences and excellent soft tissue resolution [10,11,12,13]. MRI also offers advantages in locating and staging ACCs [14]. However, to date, the relationship between the histological grade of ACCs and MR images remains unclear. Histogram analysis of apparent diffusion coefficient (ADC) values summarizes the distribution of voxel values across the whole tumor and provides more detailed quantitative information on tumor composition than traditional morphological analysis. Radiomics involves the extraction and analysis of quantitative data from medical images to characterize tumor pathology and heterogeneity, providing an opportunity for more personalized medicine. Numerous studies have indicated that radiomics demonstrates a potential role in differential diagnosis, staging, and predicting the prognosis of different tumors [15,16,17].

The current study aimed to assess whole-tumor ADC histogram analysis of diffusion-weighted imaging (DWI) for the preoperative prediction of two related pathological targets: grade III versus grade I/II ACC and the presence versus absence of a solid component. The association with overall survival was assessed as an exploratory secondary aim.

## 2. Materials and Methods

### 2.1. Subjects and Analysis of Histologic Specimens

In this study, we retrospectively reviewed consecutive patients with primary ACC of the head and neck who underwent preoperative 3.0 T MRI with DWI at our institution between January 2016 and December 2024. Inclusion criteria were (1) primary ACC of the head and neck region, (2) diagnosis confirmed by surgical pathology with histological grading, and (3) MRI examination within 2 weeks before surgery. Exclusion criteria were biopsy before MRI, because biopsy-related edema could affect the MR signal; neoadjuvant chemotherapy or radiotherapy before MRI; previous head and neck tumors or recurrent disease; and poor image quality due to artifacts. The institutional review board approved this retrospective study and waived the requirement for written informed consent. After applying the inclusion and exclusion criteria, 168 patients of 579 screened were eligible (Supplementary Figure S1). Before model development, patients were randomly allocated to a derivation cohort (*n* = 107) and an internal validation cohort (*n* = 61), with stratification by histological grade (I, II, and III) (Table 1).

All histopathological specimens were independently reviewed by two pathologists with more than 10 years of experience in head and neck tumor diagnosis, who were blinded to the clinical and imaging findings. Discrepancies were resolved by consensus or by a third senior pathologist with over 20 years of experience, and the final consensus grade was used as the reference standard. Histological grade was assigned according to the criteria of Szanto et al. [18]: grade I, tumors with tubular and cribriform areas but without solid components; grade II, cribriform tumors that were either pure or mixed with less than 30% of solid areas; and grade III, tumors with a predominantly solid pattern (solid component of 30% or more). High-grade transformation was not recorded separately. Solid-component status was defined as positive (Solid+) when any solid component was present regardless of its proportion, and as negative (Solid−) otherwise, similar to the simplified scheme proposed by van Weert et al. [19]. In this cohort, all grade II tumors contained a solid component; thus, Solid+ corresponded to grades II/III and Solid− to grade I, so the two diagnostic targets (grade III versus grades I/II, and Solid+ versus Solid−) are related endpoints.

### 2.2. MRI Scanning Protocol

To ensure consistency between cohorts, the same 3.0 T MRI unit (Ingenia, Philips Medical Systems, Best, the Netherlands) with a 16-channel head and neck coil and identical protocol was used for all patients, with monthly phantom quality assurance. Pre-enhancement MR sequences included axial T1-weighted imaging, axial and coronal T2WI, and DWI. DWI images were obtained using a spin-echo echo-planar imaging sequence with b values of 0 and 800 s/mm^2^. Axial and coronal post-enhancement T1-weighted images were obtained after administration of gadopentetate dimeglumine at 0.1 mmol/kg. Apparent diffusion coefficient (ADC) maps were constructed using a monoexponential fitting model with b values of 0 and 800 s/mm^2^. Detailed parameters were as in our previous study [20].

### 2.3. Tumor Segmentation and Feature Extraction

All MR images were digitally transferred from a picture archiving and communication system workstation to a personal computer. Tumor segmentation was performed independently by two radiologists with 5 and 7 years of experience in head and neck imaging, respectively, both blinded to the clinical and pathological data. Regions of interest (ROIs) were manually drawn along the tumor contour on each slice of the ADC maps, covering the entire tumor volume, with T2-weighted and contrast-enhanced T1-weighted images available for anatomical reference. A third radiologist with over 10 years of head and neck imaging experience reviewed the contours, and a fourth radiologist with 15 years of experience determined the final contours used for the main analysis. All segmentations were performed using ITK-SNAP 3.8.0.

**Figure 1:**
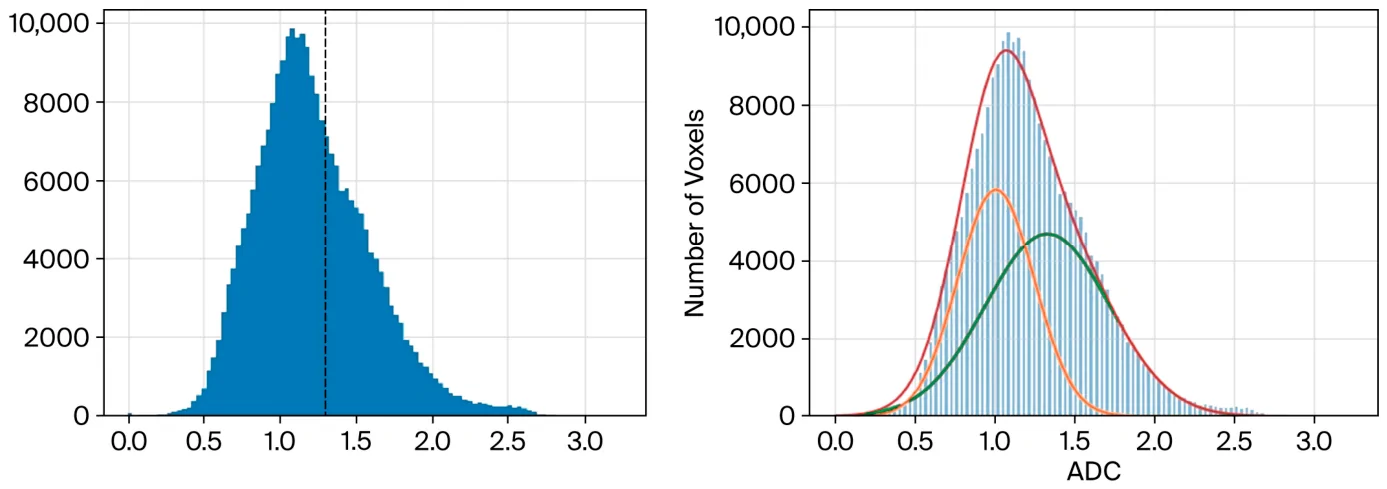
Pooled ADC distribution and illustrative Gaussian fit. The pooled histogram of apparent diffusion coefficient (ADC) values across all tumors in the derivation cohort (**left** panel) shows a unimodal, right-skewed distribution with a peak at approximately 1.15 × 10^−3^ mm^2^/s and a tail extending toward 3.0 × 10^−3^ mm^2^/s. The dashed vertical line marks the fixed Otsu threshold of 1.3 × 10^−3^ mm^2^/s used to calculate the low- and high-ADC volumes. A two-component Gaussian model fitted to the same distribution (**right** panel) is shown for illustration, with a low-ADC component (orange), a high-ADC component (green), and the total fitted distribution (red). *x*-axis, ADC (×10^−3^ mm^2^/s); *y*-axis, number of voxels.

Feature extraction used a Python-based tool built on The FeAture Explorer (FAE v0.5.5, https://github.com/salan668/FAE, accessed on 10 October 2025) engine. Otsu’s method, applied to the pooled voxelwise ADC values of the derivation cohort only (validation-cohort voxels did not contribute), yielded a fixed threshold of 1.3 × 10^−3^ mm^2^/s. This threshold was applied within each tumor ROI to calculate the volumes of voxels below and above the threshold and their fractions of the whole-tumor volume; these fractions are threshold-defined voxel fractions, not Gaussian mixture weights. Separately, a two-component Gaussian function was fitted by nonlinear least squares to a 500-bin ADC histogram of each tumor (reference implementation in Supplementary File S2) to estimate component means and standard deviations, and a pooled fit was used for illustration (Figure 1). In total, 14 histogram features were extracted and entered feature selection: (1) from the fitted Gaussian curves, the mean ADC of the lower curve (ADC_LM_), the higher curve (ADC_HM_), and the overall curve (ADC_OM_), together with their corresponding standard deviations (six parameters); (2) from the fixed threshold, the volumes of the low-ADC voxels (ADC_LV_) and the high-ADC voxels (ADC_HV_), and their relative percentages (ADC_LVP_ and ADC_HVP_) (four parameters); (3) the 10th (ADC_10_), 50th (ADC_50_), and 90th (ADC_90_) percentiles of the cumulative ADC histogram (three parameters; the nth percentile is defined as the ADC value at which n% of voxel values lie to the left); and (4) intratumoral heterogeneity (ITH), quantified as the coefficient of variation in ADC values (ITH = standard deviation/mean ADC).

Interobserver reproducibility was assessed with the features extracted from the two radiologists’ independent segmentations of all 168 tumors. Agreement was quantified with the intraclass correlation coefficient (ICC; two-way random effects, absolute agreement, single measure, ICC(A,1)) with F-based 95% confidence intervals and interpreted according to Koo and Li [21], with the lower 95% confidence limit considered alongside the point estimate. Segmentation overlap was quantified with the Dice similarity coefficient.

### 2.4. Establishment of Radiomics-Based Model and Statistical Analysis

For feature selection, redundant features were removed by Pearson correlation (|r| > 0.9), and the retained features were ranked by ANOVA F-score for discriminating grade III from grade I/II, or Solid+ from Solid−, ACC in the derivation cohort. The top two features were selected and combined in a logistic regression model. Model fitting, feature scaling, and selection of the diagnostic cutoff (maximum Youden index) were confined to the derivation cohort, and the model and cutoff were applied to the validation cohort without modification. The primary endpoint was the AUC, with the 95% CI estimated using the DeLong method and bootstrap resampling (2000 iterations). Paired AUCs were compared with the DeLong test. Optimism in the apparent AUC of the fixed two-feature signature was estimated by bootstrap resampling, and a separate exploratory selection-sensitivity analysis repeated the correlation filtering and F-score ranking in bootstrap resamples. Calibration in the validation cohort was assessed with calibration plots, the jointly estimated calibration intercept and slope, calibration-in-the-large with the slope fixed at one, and the Brier score; decision curve analysis was performed as an exploratory assessment of net benefit. To assess volume dependence, the correlation between ADCLV and total tumor volume and the variance inflation factor (VIF) were calculated, ADCLV was entered together with total tumor volume in a logistic model (both log2-transformed), and the combined model was compared with models combining ADC10 with total tumor volume or tumor diameter. Feature-by-feature comparisons between groups were exploratory; continuous variables were compared with the independent-samples t-test or, for skewed or bounded variables, the Mann–Whitney U test, and *p* values were adjusted with the Benjamini–Hochberg method within each endpoint and cohort.

The survival analysis was exploratory. Overall survival was defined as the interval from surgery to death from any cause or last follow-up, and median follow-up was estimated with the reverse Kaplan–Meier method. Metastasis was defined as nodal or distant metastasis detected on imaging during follow-up and confirmed by needle or surgical biopsy; it was recorded descriptively and was not a study endpoint. In the pooled cohort (*n* = 168), the association of the grade III diagnostic model output (per derivation-cohort standard deviation) with overall survival was evaluated in a multivariable Cox proportional hazards model adjusted for tumor diameter, age, sex, and primary site (major salivary gland versus other sites). Imaging neural invasion was not included in any model, and pathological perineural invasion and metastasis recorded during follow-up were excluded because they are not baseline preoperative variables. Histological grade and solid-component status were added separately in sensitivity models. The proportional hazards assumption was assessed with scaled Schoenfeld residuals, and a sensitivity model allowed the age effect to vary with time. In the validation cohort alone, the derivation-fitted model output and threshold were applied without modification. Kaplan–Meier curves compared exploratory groups defined by the derivation-selected threshold (log-rank test). Analyses were performed using SPSS 21.0 and Python 3.13 (scikit-learn, lifelines). All tests were two-sided (*p* < 0.05).

## 3. Results

**Figure 2:**
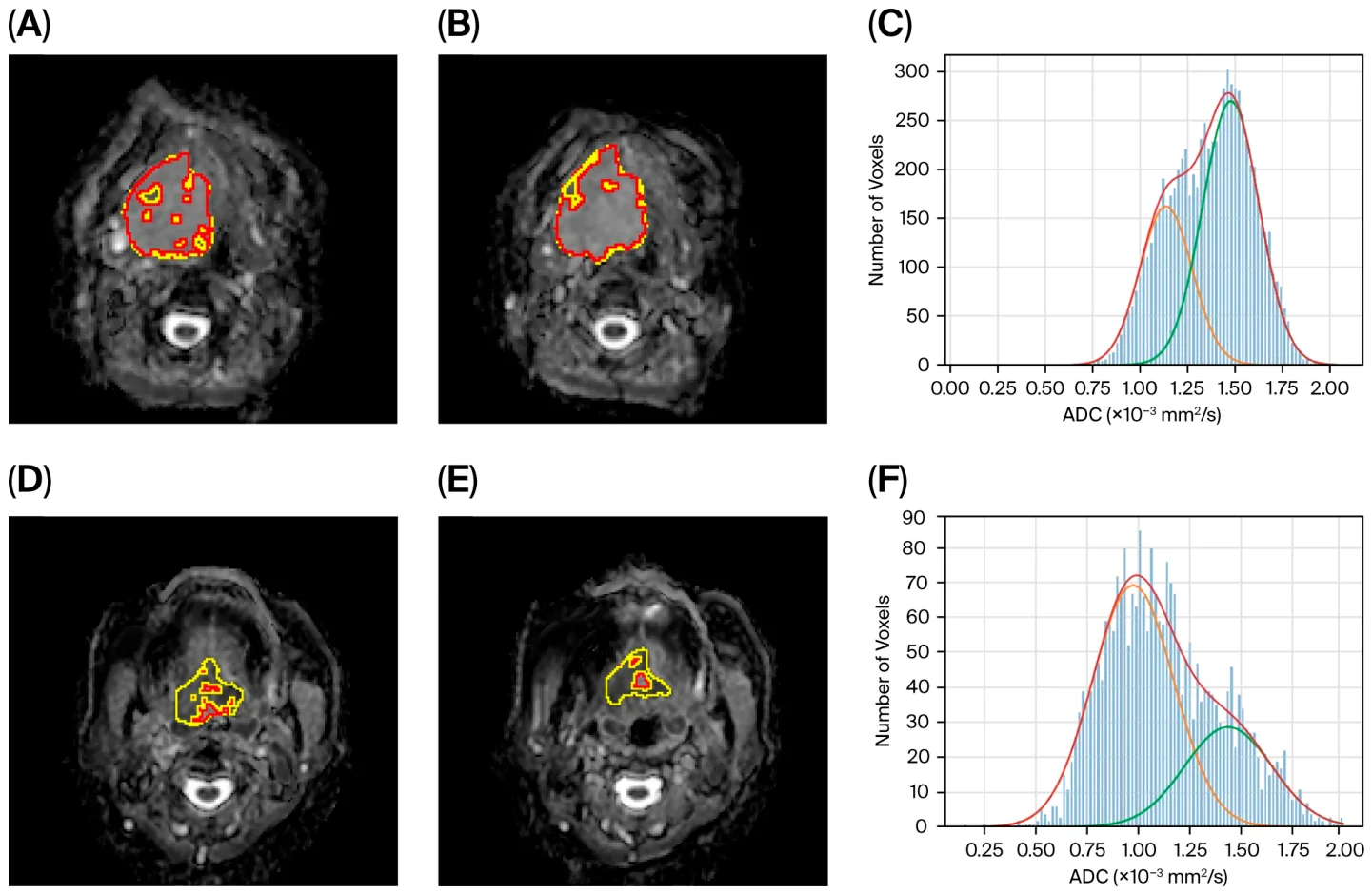
Low-ADC voxels in representative grade I and grade III tumors. (**A**,**B**) Two axial ADC maps at different levels of a 40-year-old woman with tongue ACC (histological grade I, Solid−, alive at the last follow-up 44 months after surgery). (**C**) Two-component Gaussian fit of the whole-tumor ADC histogram of the same patient. (**D**,**E**) Two axial ADC maps at different levels of a 60-year-old woman with palate ACC (histological grade III, Solid+, died 11 months after surgery). (**F**) Two-component Gaussian fit of the whole-tumor ADC histogram of the same patient. In (**A**,**B**,**D**,**E**), yellow lines outline voxels below the fixed threshold (ADC < 1.3 × 10^−3^ mm^2^/s) and red lines outline voxels at or above it; together they form the whole-tumor region of interest. In (**C**,**F**), orange = low-ADC component; green = high-ADC component; red = total fitted distribution. The grade I tumor shows a high-ADC-dominant histogram with scattered sub-threshold voxels, whereas in the grade III tumor sub-threshold voxels occupy most of the tumor, so its outer boundary appears yellow.

The pooled ADC distribution of the derivation cohort was unimodal and right-skewed (Figure 1), with a peak around 1.15 × 10^−3^ mm^2^/s and a tail extending toward 3.0 × 10^−3^ mm^2^/s. Otsu thresholding of the pooled distribution produced the fixed threshold of 1.3 × 10^−3^ mm^2^/s, which defined the low-ADC voxels (yellow outlines) within the whole-tumor ROI on ADC maps (Figure 2). A two-component Gaussian fit of the same distribution is shown for illustration (Figure 1).

### 3.1. Demographic Characteristics

From January 2016 to December 2024, 168 patients with primary ACC of the head and neck met the inclusion criteria and were enrolled. Patients were randomly allocated, with stratification by histological grade to the derivation cohort (*n* = 107) and the internal validation cohort (*n* = 61). In the derivation cohort, there were 41 men and 66 women (median age 50 years; range 25–77). In the validation cohort, there were 27 men and 34 women (median age 54 years; range 18–80). The mean tumor size was 3.6 ± 1.6 cm in the derivation cohort and 4.1 ± 2.1 cm in the validation cohort (*p* = 0.108). Major salivary gland sites accounted for 42/26 cases, other oral sites for 52/24, and sinonasal or parapharyngeal sites for 13/11 (derivation/validation). Cumulative metastasis was recorded in 100 patients (66/34), rather than being treated as a baseline covariate. There were 37 deaths (27/10). Reverse Kaplan–Meier median follow-up was 32 months in derivation, 26 months in validation, and 29 months overall (overall 95% CI 27–34). Kaplan–Meier median overall survival was 74 months overall (95% CI 68 months to an inestimable upper limit); late estimates were imprecise because only six patients remained at risk at 72 months. Numbers at risk at 24, 48, and 72 months were 95, 27, and 6 for the overall cohort; 66, 19, and 6 for Grade I, 20, 7, and 0 for Grade II, and 9, 1, and 0 for Grade III; and 66, 19, and 6 for Solid− and 29, 8, and 0 for Solid+ tumors.

### 3.2. Radiomics Parameters

**Figure 3:**
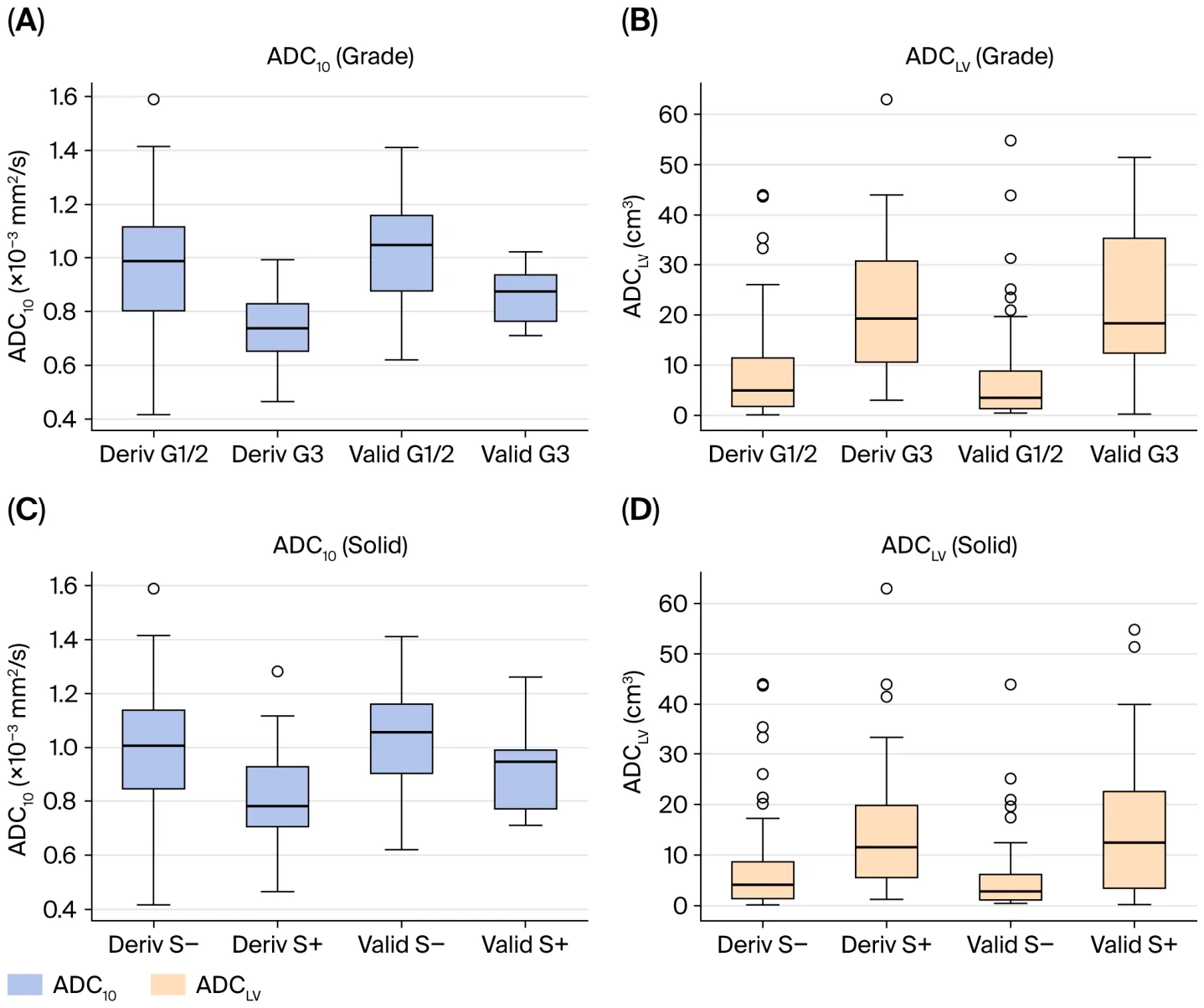
Box plots of ADC_10_ and ADC_LV_ by grade and solid status. Box plots of ADC_10_ (×10^−3^ mm^2^/s) and ADC_LV_ (cm^3^) in the derivation and validation cohorts: (**A**) ADC_10_ by histological grade, (**B**) ADC_LV_ by histological grade, (**C**) ADC_10_ by solid-component status, and (**D**) ADC_LV_ by solid-component status. ADC_10_ was significantly lower and ADC_LV_ significantly higher in grade III tumors and in Solid+ tumors than in their respective counterparts in both cohorts (all *p* < 0.01).

In the reproducibility analysis, interobserver agreement was moderate to good, with ICCs ranging from 0.755 to 0.847 and lower 95% confidence limits below 0.75 for several features (lowest, 0.502 for ITH) (Supplementary Table S1 and Supplementary Figure S2). The ICC was 0.847 (95% CI, 0.802–0.883) for ADC_10_ and 0.841 (95% CI, 0.782–0.880) for ADC_LV_, and the mean Dice similarity coefficient was 0.795 (95% CI, 0.780–0.809). The ADC histogram features in the derivation and validation cohorts are presented in Supplementary Table S2. The mean ADC values of the grade III group were significantly lower than those of the grade I/II group in the derivation cohort (all *p* < 0.01). ADC_LV_ was higher in grade III tumors in both cohorts (*p* < 0.001 and *p* = 0.009; Figure 3A,B). ITH was significantly higher in grade III than grade I/II ACC in the derivation cohort (0.228 ± 0.064 vs. 0.181 ± 0.051; *p* = 0.001), but this difference was not reproduced in the validation cohort (0.160 ± 0.078 vs. 0.165 ± 0.053; *p* = 0.778), suggesting that ITH alone may not reliably discriminate ACC histological grade. A consistent pattern was observed for solid-component status: ADC_10_ was significantly lower and ADC_LV_ significantly higher in Solid+ than in Solid− tumors in both cohorts (all *p* < 0.01; Figure 3C,D; Supplementary Table S2).

### 3.3. ROC Analyses

**Figure 4:**
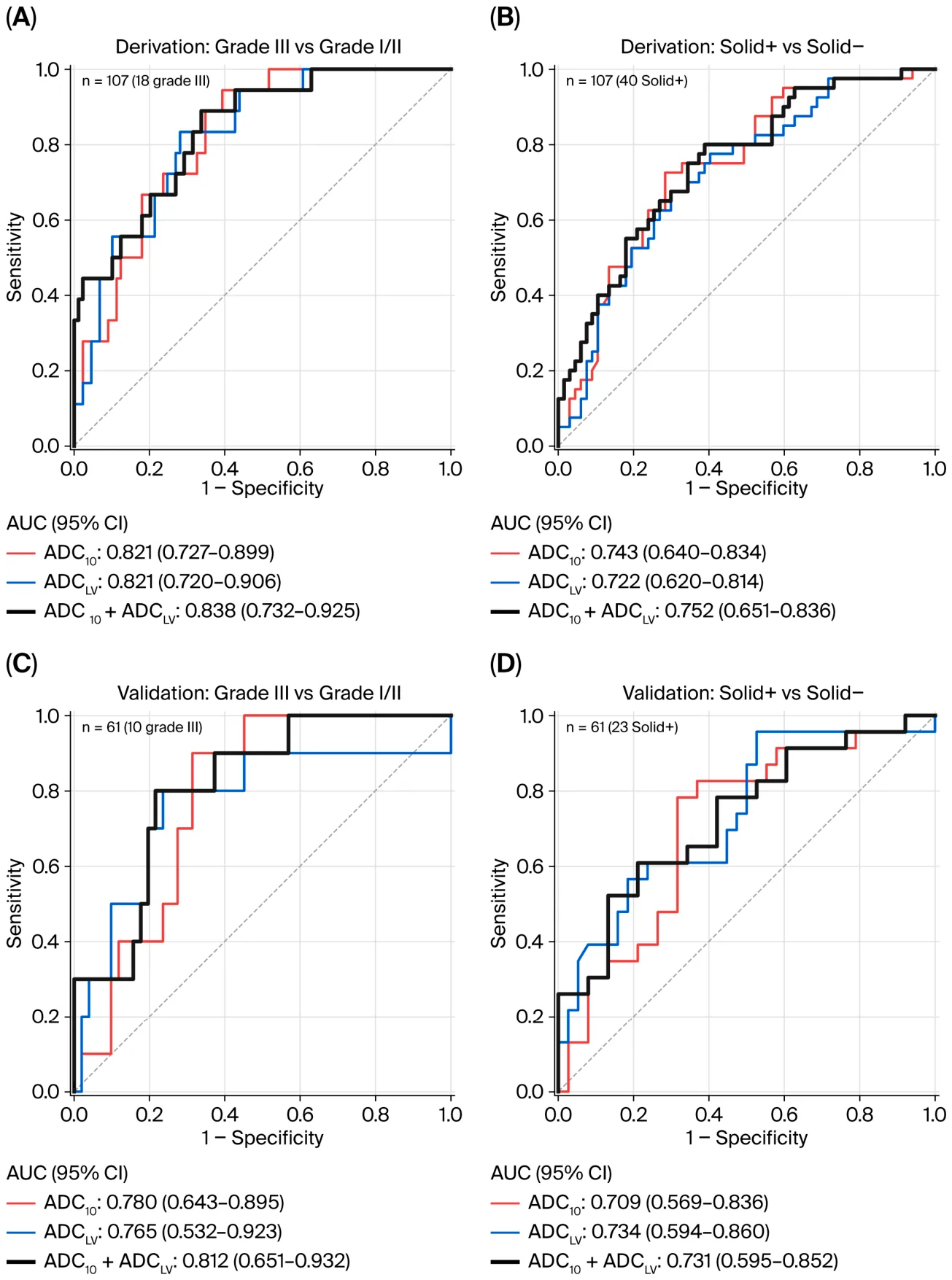
ROC curves for grade and solid-component discrimination. ROC curves of ADC_10_, ADC_LV_, and the combined ADC_10_ + ADC_LV_ model for grade III versus grade I/II (**A**,**C**) and Solid+ versus Solid− (**B**,**D**) in the derivation cohort (**A**,**B**; *n* = 107) and the internal validation cohort (**C**,**D**; *n* = 61). AUCs with 95% CIs are shown in each panel and in Table 2. The gray dashed line indicates an AUC of 0.5.

In the derivation cohort, the AUC for differentiating grade III from grade I/II ACC was 0.838 (95% CI 0.732–0.925) using the combined ADC_LV_+ADC_10_ model, compared with 0.821 (95% CI 0.727–0.899) for ADC_10_ alone, 0.821 for ADC_LV_ alone, 0.804 for ADC_50_, 0.767 for ADC_LM_, and 0.789 for ADC_OM_ (Table 2). For differentiating Solid+ from Solid− ACC, the combined model achieved an AUC of 0.752 (95% CI 0.651–0.836; Figure 4A,B). Bootstrap optimism correction changed the AUCs of the fixed two-feature signature only slightly (optimism-corrected AUC 0.833 for grade and 0.741 for solid-component status; Supplementary Figure S3), whereas the selection-sensitivity analysis gave lower AUCs (0.799 and 0.713, respectively). In the validation cohort, the locked model yielded an AUC of 0.812 (95% CI 0.651–0.932) for grade discrimination and 0.731 (95% CI 0.595–0.852) for solid-component status (Figure 4C,D). At the derivation-selected cutoff, sensitivity and specificity for grade discrimination were 80.0% and 72.5%, respectively, in the validation cohort (8 true positives, 37 true negatives, 14 false positives, and 2 false negatives). The combined model did not significantly outperform ADC_10_ alone for grade or solid-component status in the derivation cohort (DeLong *p* = 0.517 and 0.638) or in the validation cohort (*p* = 0.501 and 0.468). These non-significant differences indicate a lack of demonstrated superiority, not equivalence. Pairwise comparisons of AUC between ADC_10_ and other single ADC features were also not significant (ADC_10_ vs. ADC_50_, *p* = 0.656; ADC_10_ vs. ADC_LM_, *p* = 0.057; ADC_10_ vs. ADC_OM_, *p* = 0.393).

In the validation cohort, the calibration intercept and slope of the combined model were −0.097 (95% CI, −1.178 to 0.984) and 0.722 (95% CI, 0.245–1.198) for grade discrimination and 0.259 (95% CI, −0.467 to 0.984) and 0.953 (95% CI, 0.337–1.569) for solid-component status (Table 3). Brier scores were 0.115 for grade discrimination and 0.197 for solid-component status, compared with 0.137 and 0.235 for prevalence-only models, indicating only a limited improvement. Calibration plots and exploratory decision curves are shown in Supplementary Figure S4. In the derivation cohort, ADC_LV_ was strongly correlated with total tumor volume (Pearson r = 0.994; two-variable VIF approximately 85), and after adjustment for log2 total volume, the association of log2 ADCLV with grade III was not significant (OR, approximately 1.56; *p* = 0.523; Supplementary Figure S5). ADCLV therefore mainly reflects tumor burden rather than a distinct low-ADC compartment. Models combining ADC_10_ with total tumor volume or tumor diameter are compared with the combined model in Table 2.

### 3.4. Exploratory Survival Analysis

**Figure 5:**
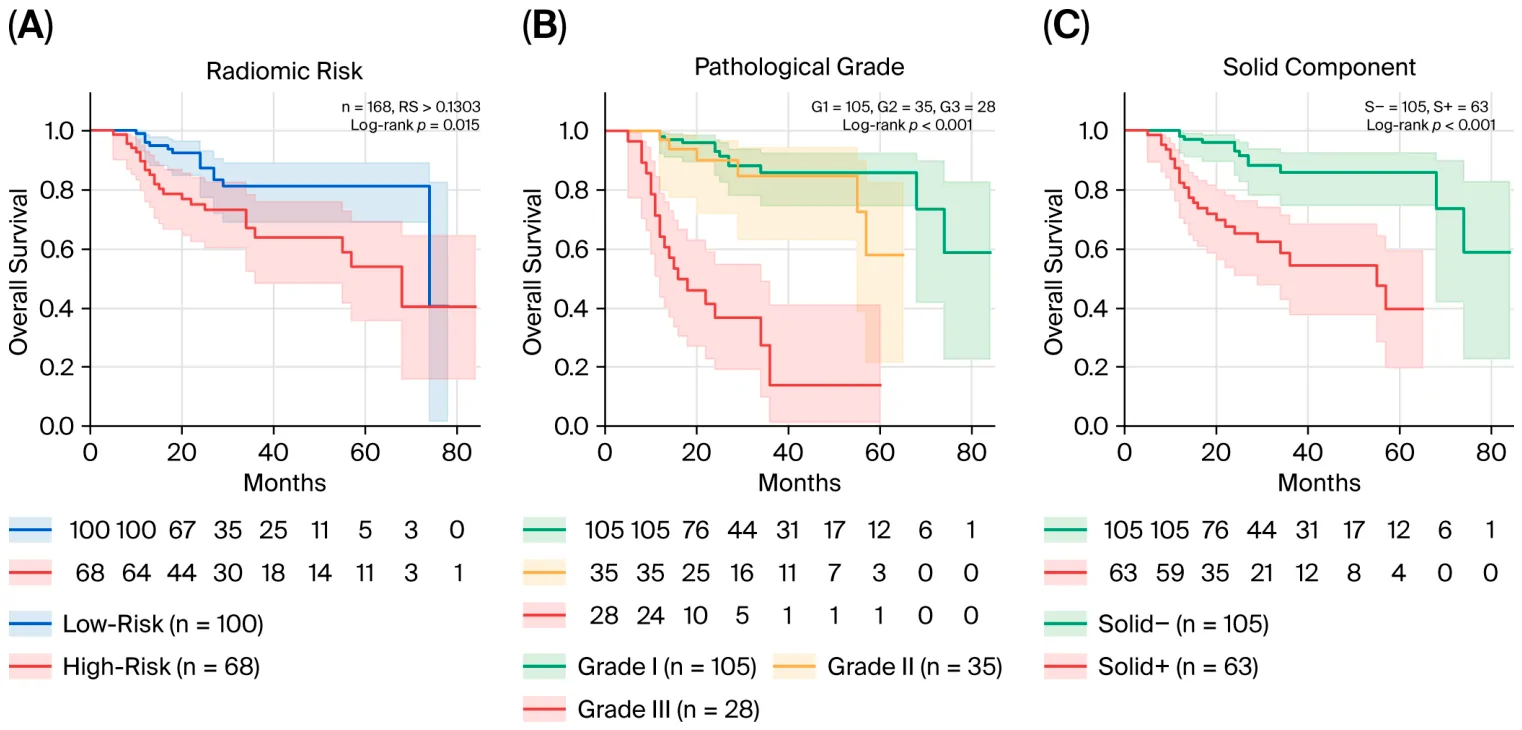
Kaplan–Meier survival curves. (**A**) Radiomic risk using the Youden-derived cutoff (RS = 0.1303): low-risk *n* = 100 vs. high-risk *n* = 68, log-rank *p* = 0.015. (**B**) Pathological grade: Grade I (*n* = 105), Grade II (*n* = 35), Grade III (*n* = 28), log-rank *p* < 0.001. (**C**) Solid-component status: Solid− (*n* = 105) vs. Solid+ (*n* = 63), log-rank *p* < 0.001. Shaded bands indicate 95% confidence intervals.

The fitted coefficients, predictor units, and derivation-cohort scaling of the diagnostic models are provided in Supplementary File S1. In the pooled cohort (*n* = 168; 37 deaths; 7.4 events per model parameter), the grade III diagnostic model output was associated with overall survival after adjustment for tumor diameter, age, sex, and primary site (HR per derivation-cohort SD, 1.627; 95% CI, 1.187–2.231; *p* = 0.002; Table 4). The model output was correlated with tumor diameter (Pearson r = 0.514; two-predictor VIF = 1.36). The association was attenuated after additional adjustment for histological grade (HR, 1.147; 95% CI, 0.795–1.653; *p* = 0.463) or solid-component status (HR, 1.40; 95% CI, 0.99–1.98; Supplementary Figure S6). In the validation cohort alone, the association was not supported (HR, 0.618; 95% CI, 0.250–1.528; *p* = 0.297; log-rank *p* = 0.895 for the fixed grouping; concordance index 0.432). The global proportional hazards test indicated nonproportionality (*p* = 0.0238), with *p* = 0.0409 for age and *p* = 0.164 for the model output; a sensitivity model allowing the age effect to vary with time gave an HR of 1.598 for the model output (95% CI, approximately 1.16–2.20; *p* = 0.004). In the pooled cohort, Kaplan–Meier analysis of the exploratory groups showed better OS in the low-risk group (14 deaths) than in the high-risk group (23 deaths; log-rank *p* = 0.015; Figure 5A). Patients with grade III tumors (log-rank *p* < 0.001; Figure 5B) and Solid+ tumors (log-rank *p* < 0.001; Figure 5C) had worse OS. Numbers at risk for each group are shown below each panel of Figure 5.

## 4. Discussion

In this study, whole-tumor ADC histogram analysis was used to develop a noninvasive approach for characterizing adenoid cystic carcinoma before treatment. The two derived parameters, ADC_10_ and the low-ADC component volume (ADC_LV_), were associated with a solid growth component and high histological grade. Because histological grade is a major determinant of prognosis in ACC, a preoperative imaging-based estimate of these features may be of potential clinical value. Nevertheless, ADC_LV_ was almost collinear with total tumor volume (r = 0.994), so the combined model mainly reflects ADC_10_ plus tumor size; moreover, no treatment-decision or prospective clinical-utility analysis was performed, and the model should be regarded as a potentially useful preoperative research tool rather than a basis for deciding the extent of resection or the intensity of treatment. However, the combined model did not significantly outperform ADC_10_ alone, and the exploratory association with overall survival was not supported in the validation cohort or after adjustment for histological grade.

One of the biological characteristics of ACC is slow but relentless growth and dissemination [5,7]. Treatment of ACC is complicated and can be influenced by the location of the tumor, stage at diagnosis, and biologic behavior as reflected in histologic grade [1,2,4]. Theoretically, the best treatment for an ACC tumor that is deemed potentially resectable after extensive workup is radical surgical resection, ensuring negative margins, and adjuvant radiotherapy [5,6,7,8,22]. However, surgical resection of a tumor with a negative margin is impossible in some cases, which leaves radiotherapy or chemotherapy as the only treatment choice [6]. Additionally, ACC is characterized by slow progression and late onset of distant metastases, which sometimes leads to treatment failure.

Many studies have reported different preoperative factors that influence the prognosis of ACC, including primary site, size, bone and nerve invasion, metastatic lymph nodes, and histologic pattern [5,7,8,22,23,24,25]. Of these factors, histologic pattern appears to be most closely correlated with the prognosis of ACC [5,7]. However, there is still no method to accurately determine the histological grade of ACC before operation. Radiomics is becoming more popular and widely used in disease diagnosis, but many image features lack a clear biological interpretation.

Due to excellent soft tissue resolution, preoperative MRI has an advantage in locating and staging head and neck tumors [14,20]. DWI can help characterize some head and neck tumors, especially salivary gland tumors [10,11,12,13], mainly because ADC is inversely related to tissue cellularity. ADC-based radiomic features have also been applied in other head and neck tumors, for example, to diagnose local recurrence of squamous cell carcinoma [26]. Within ACC, the pseudocystic spaces of tubular and cribriform areas contain basophilic mucoid and hyalinized basement-membrane-like material, which, together with the hyalinized stroma, may explain their relatively high ADC, whereas the solid pattern consists of sheets of basaloid cells with pleomorphism and frequent mitoses and is expected to show a lower ADC. Necrosis, including comedonecrosis in solid areas, may raise ADC and complicate this correspondence. This also implies that single-sequence DWI has inherent limitations: multisequence MRI analysis, including contrast-enhanced imaging to delineate necrotic regions, is warranted to improve the specificity of ADC-based tissue characterization.

Because the three growth patterns frequently coexist within a single lesion, ADC values—whether averaged over the whole tumor or biased by the choice of region of interest—may not capture this heterogeneity. Histogram features describe the distribution of ADC values across the whole tumor, and MRI-based radiomic features have been studied for the characterization of salivary gland tumors [13]. The present features summarize the ADC distribution and do not quantify the spatial arrangement of tumor subregions; the low-ADC voxel overlays in Figure 1 and Figure 2 illustrate the processing approach. The association between histogram features and grade is therefore plausible but has not been validated against voxel-matched histology.

We also explored the association between diagnostic model output and overall survival. Although an association was observed in the pooled cohort, it was not supported in the validation cohort, and it was attenuated after adjustment for histological grade; moreover, the model output contains a volume-related feature and was correlated with tumor diameter. The output may therefore act mainly as a preoperative surrogate of grade and tumor burden rather than as an independent prognostic marker, and these survival findings should be regarded as exploratory. This lack of consistent prognostic validation is one of the factors currently limiting clinical application of the model. In addition, the median follow-up of 29 months is short for an indolent malignancy characterized by late recurrence and metastasis, and only overall survival could be analyzed.

This study has several limitations. First, although this is the largest series of ACCs evaluated using DWI-based histogram analysis, the overall sample size remains modest (*n* = 168), reflecting the low clinical incidence of this tumor, and the validation cohort contained only 10 grade III and 23 Solid+ tumors, which limits the precision of discrimination and calibration estimates. Second, the cohorts were formed using grade-stratified random allocation within a single center, scanner, and protocol; this internal holdout validation does not assess temporal or external generalizability. Third, the features were selected in a small derivation cohort, and the selection-sensitivity analysis indicated that selection uncertainty lowers the expected performance. In addition, the calibration slope of 0.722 (95% CI 0.245–1.198) is consistent with optimism. Fourth, the fixed threshold was derived from pooled derivation-cohort voxels, which gives larger tumors more weight, although the derivation AUC for grade III remained 0.83–0.85 across thresholds of 1.1–1.5 × 10^−3^ mm^2^/s. Fifth, ADC was calculated from two b values (0 and 800 s/mm^2^) and therefore includes a perfusion-related contribution, and echo-planar DWI is prone to susceptibility-related distortion at sinonasal and skull-base sites. Sixth, patients who underwent biopsy before MRI were excluded, which may limit generalizability, particularly for accessible oral sites where preoperative biopsy is routine. Seventh, high-grade transformation was not recorded separately; transformed tumors, if present, would probably have been classified as grade III and, because of their high cellularity, could inflate the apparent discrimination of grade III [7]. In addition, the high cumulative metastasis rate (59.5%) suggests a more aggressive case mix than in most published series, which may limit generalizability. Finally, T and N categories, margin status, treatment details, and the dates of recurrence or metastasis were not available, so complete adjustment and analyses of local recurrence-free, distant metastasis-free, or disease-specific survival were not possible.

In conclusion, among 14 ADC histogram features extracted, ADC_LV_ and ADC_10_ were identified through data-driven feature selection as the strongest discriminators of ACC histological grade, although ADCLV largely reflects tumor volume. A logistic regression model combining these two features showed promising discrimination in the derivation cohort (AUC = 0.838, 95% CI 0.732–0.925) and in a grade-stratified random internal validation cohort (AUC = 0.812, 95% CI 0.651–0.932). However, ADC10 alone achieved comparable performance (AUCs of 0.821, 95% CI 0.727–0.899, and 0.780, 95% CI 0.643–0.895, for grade III in the derivation and validation cohorts, and 0.743 and 0.709 for solid-component status), and adding the low-ADC volume did not improve discrimination (DeLong *p* ≥ 0.468). The exploratory association with overall survival was not supported in the validation cohort, so the prognostic value of the model remains unconfirmed. Multicenter external validation with larger cohorts is warranted.

## Figures and Tables

**Table 1:** Characteristics and follow-up of the derivation and internal validation cohorts.

Variable	Derivation *n* = 107	Validation *n* = 61	*p* Value
Age, years, median (IQR)	50 (42.5–59.5)	54 (47–63)	0.099
Male/female, *n*	41/66	27/34	0.514
Diameter, cm, mean ± SD	3.6 ± 1.6	4.1 ± 2.1	0.108
Grade I/II/III, *n*	67/22/18	38/13/10	0.992
Solid−/Solid+, *n*	67/40	38/23	1.000
Major salivary gland, *n*	42	26	0.409
Other oral site, *n*	52	24	
Sinonasal/parapharyngeal, *n*	13	11	
Pathological nerve invasion, *n* (%)	88 (82.2)	49 (80.3)	0.837
Cumulative metastasis, *n* (%)	66 (61.7)	34 (55.7)	0.514
Deaths, *n* (%)	27 (25.2)	10 (16.4)	0.245
Reverse-KM follow-up, months	32	26	—

Follow-up was estimated using the reverse Kaplan–Meier method. Metastasis was recorded at any time during follow-up and is not a baseline variable. χ^2^ test across the three site groups. IQR, interquartile range; SD, standard deviation.

**Table 2:** Discrimination of the diagnostic models in the derivation and internal validation cohorts.

Model	Grade III vs. Grade I/II		Solid+ vs. Solid−	
	Derivation (*n* = 107)	Validation (*n* = 61)	Derivation (*n* = 107)	Validation (*n* = 61)
ADC_10_ + ADC_LV_ (combined)	0.838 (0.732–0.925)	0.812 (0.651–0.932)	0.752 (0.651–0.836)	0.731 (0.595–0.852)
ADC_10_	0.821 (0.727–0.899)	0.780 (0.643–0.895)	0.743 (0.640–0.834)	0.709 (0.569–0.836)
ADC_LV_	0.821 (0.720–0.906)	0.765 (0.532–0.923)	0.722 (0.620–0.814)	0.734 (0.594–0.860)
ADC_10_ + total tumor volume	0.829 (0.723–0.916)	0.767 (0.610–0.892)	0.743 (0.637–0.839)	0.709 (0.568–0.835)
ADC_10_ + tumor diameter	0.827 (0.722–0.913)	0.788 (0.656–0.896)	0.743 (0.635–0.835)	0.704 (0.560–0.832)
*P*, combined vs. ADC_10_	0.517	0.501	0.638	0.468
Optimism-corrected AUC, combined	0.833	—	0.741	—

Values are AUC (95% CI). 95% CIs were estimated by bootstrap resampling (2000 iterations); *p* values are from paired DeLong tests. AUC, area under the receiver operating characteristic curve; CI, confidence interval.

**Table 3:** Calibration of the combined model in the internal validation cohort (*n* = 61).

Endpoint	Calibration Intercept (95% CI)	Calibration Slope (95% CI)	Calibration-in-the-Large	Brier Score
Grade III vs. grade I/II	−0.097 (−1.178 to 0.984)	0.722 (0.245–1.198)	0.318	0.115
Solid+ vs. Solid−	0.259 (−0.467 to 0.984)	0.953 (0.337–1.569)	0.293	0.197

Intercept and slope were estimated jointly; calibration-in-the-large was estimated with the slope fixed at one. The validation cohort contained 10 grade III and 23 Solid+ tumors. CI, confidence interval.

**Table 4:** Multivariable Cox regression of overall survival for the grade III diagnostic model output (pooled cohort, *n* = 168; 37 deaths).

Variable	HR (95% CI)	*p*
Diagnostic model output (per derivation-cohort SD)	1.627 (1.187–2.231)	0.002
Tumor diameter (per cm)	1.09 (0.87–1.36)	0.439
Age (per year)	1.01 (0.98–1.03)	0.607
Sex (male vs. female)	2.13 (1.10–4.14)	0.025
Primary site (major salivary gland vs. other)	0.77 (0.37–1.58)	0.476
Sensitivity analyses: HR of the diagnostic model output		
Additionally adjusted for histological grade	1.147 (0.795–1.653)	0.463
Additionally adjusted for solid-component status	1.40 (0.99–1.98)	0.054
Validation cohort only (*n* = 61; 10 deaths)	0.618 (0.250–1.528)	0.297

Main model: 37 deaths for five parameters (7.4 events per parameter). Proportional hazards: global *p* = 0.0238; age, *p* = 0.0409; model output, *p* = 0.164. Imaging neural invasion, pathological perineural invasion, and metastasis during follow-up were not included. HR, hazard ratio; SD, standard deviation.

## Data Availability

The data presented in this study are available on reasonable request from the corresponding author.

## Supplementary Materials

The following supporting information can be downloaded at https://www.mdpi.com/article/10.3390/diagnostics16213452/s1, Figure S1: Flow diagram of patient selection; Figure S2: Interobserver agreement of the 14 ADC histogram features; Figure S3: Apparent and optimism-corrected AUCs; Figure S4: Calibration plots and decision curves in the internal validation cohort; Figure S5: Low-ADC volume versus total tumor volume; Figure S6: Hazard ratios of the diagnostic model output across survival models; Table S1: Interobserver reproducibility of the ADC histogram features and tumor segmentation; Table S2: ADC histogram features by histological grade and solid-component status; File S1: Fitted model parameters (model_parameters.json); File S2: Reference implementation of the two-component Gaussian histogram fit (Python script).

## Abbreviations

ACC, adenoid cystic carcinoma; ADC, apparent diffusion coefficient; AUC, area under the ROC curve; CI, confidence interval; DWI, diffusion-weighted imaging; HR, hazard ratio; ICC, intraclass correlation coefficient; ITH, intratumoral heterogeneity; IQR, interquartile range; MRI, magnetic resonance imaging; OR, odds ratio; OS, overall survival; ROC, receiver operating characteristic; ROI, region of interest; SD, standard deviation; VIF, variance inflation factor.

## References

[1] Dewenter I., Otto S., Kakoschke T.K., Smolka W., Obermeier K.T. (2023). Recent Advances, Systemic Therapy, and Molecular Targets in Adenoid Cystic Carcinoma of the Head and Neck. J. Clin. Med..

[2] Saleh E., Ukwas A. (2023). Adenoid Cystic Carcinoma of Salivary Glands: A Ten-Year Review and an Assessment of the Current Management, Surgery, Radiotherapy, and Chemotherapy. Int. J. Otolaryngol..

[3] Conley J., Dingman D.L. (1974). Adenoid Cystic Carcinoma in the Head and Neck (Cylindroma). Arch. Otolaryngol..

[4] de Morais E.F., de Farias Morais H.G., de Almeida Freitas R., Coletta R.D. (2023). Prognostic Significance of Histopathological Parameters for Salivary Gland Adenoid Cystic Carcinoma. Dent. J..

[5] Jia Y., Liu Y., Yang H., Yao F. (2024). Adenoid Cystic Carcinoma: Insights from Molecular Characterization and Therapeutic Advances. MedComm.

[6] Lorini L., Ardighieri L., Bozzola A., Romani C., Bignotti E., Buglione M., Guerini A., Lombardi D., Deganello A., Tomasoni M. (2021). Prognosis and Management of Recurrent and/or Metastatic Head and Neck Adenoid Cystic Carcinoma. Oral Oncol..

[7] Zhu Y., Zhu X., Xue X., Zhang Y., Hu C., Liu W., Lu H. (2021). Exploration of High-Grade Transformation and Postoperative Radiotherapy on Prognostic Analysis for Primary Adenoid Cystic Carcinoma of the Head and Neck. Front. Oncol..

[8] Cheng Y., Xu L., Chen Z., Wu H., Zou H., Zhang T., Liu G., Liu Z., Yin C., Ma L. (2023). Prognosis of Adenoid Cystic Carcinoma in Head and Neck Region Treated with Different Regimens—A Single-Centre Study. Cancer Med..

[9] Almulla L.A., Alnujaidi A.M., Alhezam Y.I., Slais H.A., Mosaqlab M.A., Khuraidah H.A., Awadalla A., Alsayyah A. (2025). Fine Needle Aspiration vs. Surgical Pathology Specimen in Salivary Gland Tumors: A Study of Diagnostic Accuracy and Predictive Value. Ann. Saudi Med..

[10] Gökçe E., Beyhan M. (2022). Advanced Magnetic Resonance Imaging Findings in Salivary Gland Tumors. World J. Radiol..

[11] Nagano S.Y.M., Chojniak R., Porto G.C.L.M. (2025). Magnetic Resonance Permeability for the Evaluation of Head and Neck Tumors: Parotid and Beyond. J. Clin. Imaging Sci..

[12] Maraghelli D., Pietragalla M., Cordopatri C., Nardi C., Peired A.J., Maggiore G., Colagrande S. (2021). Magnetic Resonance Imaging of Salivary Gland Tumours: Key Findings for Imaging Characterisation. Eur. J. Radiol..

[13] Mao K., Wong L.M., Zhang R., So T.Y., Shan Z., Hung K.F., Ai Q.Y.H. (2023). Radiomics Analysis in Characterization of Salivary Gland Tumors on MRI: A Systematic Review. Cancers.

[14] Wang Y., Guo X., Yu K., Shen X., Liu J., Zhao T., Gu H. (2023). Adenoid Cystic Carcinoma of Head and Neck: Summary and Review of Imaging Findings. Heliyon.

[15] Rao Y., Ma Y., Wang J., Xiao W., Wu J., Shi L., Guo L., Fan L. (2024). Performance of Radiomics in the Differential Diagnosis of Parotid Tumors: A Systematic Review. Front. Oncol..

[16] Korte J.C., Cardenas C., Hardcastle N., Kron T., Wang J., Bahig H., Elgohari B., Ger R., Court L., Fuller C.D. (2021). Radiomics Feature Stability of Open-Source Software Evaluated on Apparent Diffusion Coefficient Maps in Head and Neck Cancer. Sci. Rep..

[17] Rodrigues A., Loman K., Nawrocki J., Hoang J.K., Chang Z., Mowery Y.M., Oyekunle T., Niedzwiecki D., Brizel D.M., Craciunescu O. (2021). Establishing ADC-Based Histogram and Texture Features for Early Treatment-Induced Changes in Head and Neck Squamous Cell Carcinoma. Front. Oncol..

[18] Szanto P.A., Luna M.A., Tortoledo M.E., White R.A. (1984). Histologic Grading of Adenoid Cystic Carcinoma of the Salivary Glands. Cancer.

[19] van Weert S., van der Waal I., Witte B.I., Leemans C.R., Bloemena E. (2015). Histopathological Grading of Adenoid Cystic Carcinoma of the Head and Neck: Analysis of Currently Used Grading Systems and Proposal for a Simplified Grading Scheme. Oral Oncol..

[20] Tang W., Wang Y., Yuan Y., Tao X. (2022). Assessment of Tumor Depth in Oral Tongue Squamous Cell Carcinoma with Multiparametric MRI: Correlation with Pathology. Eur. Radiol..

[21] Koo T.K., Li M.Y. (2016). A Guideline of Selecting and Reporting Intraclass Correlation Coefficients for Reliability Research. J. Chiropr. Med..

[22] Scarpa A., Viola P., Ralli M., Gioacchini F.M., Salzano G., Di Stadio A., Cassandro C., Chiarella G., Ricciardiello F., De Luca P. (2024). Post-Operative Radiotherapy in Adenoid Cystic Carcinoma of Salivary Glands versus Surgery Alone: What Is the Evidence about Survival and Local Control? A Systematic Review and Meta-Analysis. Eur. Arch. Otorhinolaryngol..

[23] Zupancic M., Näsman A., Berglund A., Dalianis T., Friesland S. (2023). Adenoid Cystic Carcinoma (AdCC): A Clinical Survey of a Large Patient Cohort. Cancers.

[24] Gan J.Y., Nguyen K., Elliott M.S., Palme C.E., Shannon K., Clark J., Wykes J., Gupta R., Low H.T. (2025). Prognostic Factors Affecting Survival in Patients with Head and Neck Adenoid Cystic Carcinoma: A Single-Institution Experience. Cureus.

[25] Lavareze L., Kimura T.D.C., Cacita N., de Lima-Souza R.A., Cattan M.E.S., Egal E.S.A., Altemani A., Mariano F.V. (2025). Survival Outcomes in Adenoid Cystic Carcinoma of the Head and Neck: A Systematic Review of 17,497 Cases and Meta-Analysis. Head Neck.

[26] Kim M., Lee J.H., Joo L., Jeong B., Kim S., Ham S., Yun J., Kim N., Chung S.R., Choi Y.J. (2022). Development and Validation of a Model Using Radiomics Features from an Apparent Diffusion Coefficient Map to Diagnose Local Tumor Recurrence in Patients Treated for Head and Neck Squamous Cell Carcinoma. Korean J. Radiol..

