# Supplementary material for: Apparent Diffusion Coefficient Histogram Analysis of Adenoid Cystic Carcinoma of the Head and Neck: Correlation with Histological Grade and Exploratory Survival Analysis
Source: Diagnostics (Basel). 2026 Oct 22;16(21):3452. doi: 10.3390/diagnostics16213452 (PMC13761860; doi:10.3390/diagnostics16213452)
Supplement: Supplementary file 1 [file diagnostics-16-03452-s001.zip › Supplementary_Figure_Legends.pdf]

Supplementary Figure Legends

Figure S1. Flow diagram of patient selection. Of 579 patients screened, 411 were excluded (no surgical resection, n = 39; recurrent disease, n = 102; CT only, n = 205; DWI not acquired with the study protocol, n = 27; prior treatment, n = 34; poor image quality, n = 4). The 168 included patients were randomly allocated, with stratification by histological grade, to the derivation cohort (n = 107; grade I/II/III, 67/22/18) and the internal validation cohort (n = 61; 38/13/10).

Figure S2. Interobserver agreement of the 14 candidate ADC histogram features. Dots show the intraclass correlation coefficient (ICC; two-way random effects, absolute agreement, single measure) between features extracted from the two observers' independent segmentations of 168 tumors, and horizontal lines show the F-based 95% confidence intervals. Shaded bands mark the reliability categories of Koo and Li (poor, < 0.50; moderate, 0.50–0.75; good, 0.75–0.90; excellent, > 0.90). ICCs ranged from 0.755 to 0.847. Numerical values and bootstrap confidence intervals are listed in Supplementary Table S1.

Figure S3. Apparent and optimism-corrected AUCs in the derivation cohort. (A) Grade III versus grade I/II; (B) Solid+ versus Solid−. Blue, fixed two-feature signature (ADC10 + ADCLV) with bootstrap optimism correction; orange, exploratory selection-sensitivity analysis in which correlation filtering and F-score ranking were repeated in bootstrap resamples. AUC, area under the receiver operating characteristic curve.

Figure S4. Calibration and decision curves of the combined model in the internal validation cohort (n = 61). (A, B) Calibration plots for grade III versus grade I/II (A) and Solid+ versus Solid− (B); points show the observed proportion within quintiles of predicted probability with Wilson 95% confidence intervals, and the dotted line indicates perfect calibration. (C, D) Exploratory decision curves for the same endpoints, compared with treat-all and treat-none strategies. Calibration estimates are listed in Table 3.

Figure S5. Low-ADC volume versus total tumor volume in the derivation cohort (n = 107). Each point represents one tumor. The dotted line is the line of identity, and the solid line is the least-squares fit. Low-ADC volume is the volume of voxels below the fixed threshold of 1.3×10^{-3} mm^2/s; total volume is the sum of the low- and high-ADC voxel volumes. r, Pearson correlation coefficient.

Figure S6. Hazard ratios of the grade III diagnostic model output for overall survival across exploratory Cox models. Points show the hazard ratio per derivation-cohort standard deviation of the model output, with 95% confidence intervals, for the main model adjusted for preoperative covariates (n = 168), the model adjusted for tumor size, the models additionally adjusted for histological grade or solid component status, and the validation cohort alone (n = 61). The dotted line marks a hazard ratio of 1.
